# Interrogating stonefish venom: small molecules present in envenomation caused by *Synanceia* spp.

**DOI:** 10.1002/2211-5463.13926

**Published:** 2024-11-20

**Authors:** Silvia Luiza Saggiomo, Steve Peigneur, Jan Tytgat, Norelle L. Daly, David Thomas Wilson

**Affiliations:** ^1^ Australian Institute of Tropical Health and Medicine James Cook University Cairns Australia; ^2^ Toxicology and Pharmacology Katholieke Universiteit (KU) Leuven Belgium; ^3^ Present address: Hepatic Fibrosis Group QIMR Berghofer Medical Research Institute Brisbane Australia

**Keywords:** acetylcholine, GABA, stonefish, *Synanceia*, venom

## Abstract

The stonefish *Synanceia verrucosa* and *Synanceia horrida* are arguably the most venomous fish species on earth and the culprits of severe stings in humans globally. Investigation into the venoms of these two species has mainly focused on protein composition, in an attempt to identify the most medically relevant proteins, such as the lethal verrucotoxin and stonustoxin components. This study, however, focused on medically relevant small molecules, and through nuclear magnetic resonance, mass spectroscopy, and fractionation techniques, we discovered and identified the presence of three molecules new to stonefish venom, namely γ‐aminobutyric acid (GABA), choline and *0*‐acetylcholine, and provide the first report of GABA identified in a fish venom. Analysis of the crude venoms on human nicotinic acetylcholine receptors (nAChRs) and a GABA_A_ receptor (GABA_A_R) showed *S. horrida* venom could activate neuronal (α7) and adult muscle‐type (α1β1δε) nAChRs, while both crude *S. horrida* and *S. verrucosa* venoms activated the GABA_A_R (α1β2γ2). Cytotoxicity studies in immunologically relevant cells (human PBMCs) indicated the venoms possess cell‐specific cytotoxicity and analysis of the venom fractions on Na^+^ channel subtypes involved in pain showed no activity. This work highlights the need to further investigate the small molecules found in venoms to help understand the mechanistic pathways of clinical symptoms for improved treatment of sting victims, in addition to the discovery of potential drug leads.

## Graphical abstract

Molecular analysis of *Synanceia horrida* (ShV) and *S. verrucosa* (SvV) venoms identified three small molecules new to stonefish venom: Gamma‐aminobutyric acid (GABA), choline, and *0*‐acetylcholine. Analysis on human nicotinic acetylcholine (nAChRs) and GABA_A_ receptors showed activation of neuronal (α7) and adult muscle‐type (α1β1δε) nAChRs by ShV, while both ShV and SvV activated GABA_A_R (α1β2γ2).

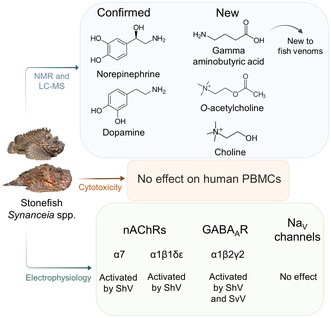

**Abbreviations**
1D: one‐dimensional
2D: two‐dimensional
ACh: acetylcholine
COSY: correlated spectroscopy
DA: dopamine
DNA: deoxyribonucleic acid
DPBS: Dulbecco's phosphate‐buffered saline
FA: formic acid
GABA: gamma‐aminobutyric acid
GABA_A_R: gamma‐aminobutyric acid type A receptor
His: histamine
HMBC: heteronuclear multiple bond correlation
hPBMCs: human peripheral blood mononuclear cells
HSQC: heteronuclear single quantum coherence
LC–MS: liquid chromatography‐mass spectrometry
nAChRs: nicotinic acetylcholine receptors
Na_v_ Channel: voltage‐gated sodium channel
NE: norepinephrine
NMR: nuclear magnetic resonance
NOESY: nuclear overhauser effect spectroscopy
RP‐HPLC: reversed‐phase high performance liquid chromatography
RPMI: Roswell Park Memorial Institute
SDS/PAGE: sodium dodecyl sulfate/polyacrylamide
SEC: size‐exclusion chromatography
ShV: *S. horrida* venom
SPE: solid‐phase extraction
SvV: *S. verrucosa* venom
TIC: total ion current
TOCSY: total correlation spectroscopy
Trp: tryptophan
UV_280 nm_
: ultraviolet light at 280 nanometers

Several venomous fish species produce painful and potentially lethal human envenomation. The Synanceiidae family, which incorporates the estuarine (*Synanceia horrida*) and reef stonefish (*Synanceia verrucosa*), is of particular note. These are regarded as the world's most venomous fish species [1] and are found throughout the warm waters of the Indo‐Pacific region and also in the Persian Gulf and the Red Sea [2]. As with many venomous fish, the venom apparatus present in the Synanceiidae family lacks musculature, so envenomation happens involuntarily as a defense mechanism [3]. Upon envenomation, severe pain can radiate from the affected limb to the regional lymphatics, where oedema and erythema may happen. Apart from these localized symptoms, systemic complications may develop, such as weakness, tachycardia, pulmonary oedema, convulsions, respiratory and/or cardiac failure [2], and death [4].

The mechanisms through which stonefish venoms give rise to the observed pathologies and symptoms still remain poorly understood. Venoms are generally composed of many different proteins, peptides, salts, and small molecules, which can act in synergy to produce toxic effects [5]. Characterization of these molecules can provide insight into the ecology of the animals, envenomation treatment, and also serve as inspiration for drug development, with several examples of venom‐derived compounds in preclinical or clinical development, or being used as marketed drugs [6, 7, 8]. Despite the medical importance of stonefish, there is still much to be characterized in terms of venom composition. Several compounds have been identified, which include large proteinaceous toxins such as verrucotoxin and stonustoxin, responsible for some of the lethal, haemolytic, neurotoxic, cytolytic, cardiotoxic, vasorelaxant, and inflammatory activities [9, 10, 11, 12, 13, 14]. Cardioleputin, a 46 kDa protein that causes chronotropic and inotropic effects [15], a 45 kDa lectin that causes agglutination in rabbit erythrocytes [16], a cytolysin [17], and enzymes such as hyaluronidases, acetylcholinesterase, and phosphodiesterase have also been identified [18, 19, 20, 21]. In terms of small molecules, dopamine, norepinephrine, tryptophan, and histamine have been reported [22, 23]. However, a recent proteomic and transcriptomic analysis of components found in *S. horrida* venom also indicated the presence of numerous uncharacterised and unique toxins [24], which highlights the need for further investigation into stonefish venoms.

The present work explores components found in *S. verrucosa* and *S. horrida* venoms to further characterize their molecular composition. This was accomplished through the use of nuclear magnetic resonance spectroscopy, liquid chromatography‐mass spectrometry, and venom fractionation. Molecules not previously reported in fish venoms were discovered and identified, and the implications of these findings are discussed. We hope this manuscript inspires a search for molecules other than proteins and enzymes to better understand envenomation mechanisms.

## Materials and methods

### Animal collection

Two different species of stonefish, *S. verrucosa* and *S. horrida*, were used in the experiments (ethics number A2572, James Cook University Animal Ethics Committee). Individuals were housed together and apart from other fish species at James Cook University Aquarium. Hereinafter, *S. verrucosa* venom will be referred to as SvV and *S. horrida* venom will be referred to as ShV.

### Venom collection

Venom was collected from either all of the dorsal spines or from a selected number. Milking was performed with at least a four‐week interval between milking events, based on venom regeneration times [25]. Venom collection was performed using a tube containing a membranous lid as previously reported [26]. Venom was immediately placed in an ice bath, centrifuged at 4 °C for 10 min at 20,627 gto remove particulates, and used for experiments as crude fresh venom or divided into aliquots, frozen, or lyophilized, and stored at −80 °C. Venom protein concentrations were assayed using a bicinchoninic acid (BCA) protein assay kit according to the manufacturer's instructions (Pierce Rapid Gold, ThermoFisher Scientific, Brisbane, Queensland, Australia; Cat A53225). Frozen venom was allowed to thaw in an ice bath and lyophilized venom was rehydrated in Dulbecco's phosphate‐buffered saline (DPBS, ThermoFisher) prior to usage.

### Analytical assessment of crude venoms

#### SDS‐PAGE gel

Frozen SvV and lyophilized ShV were used for this experiment. SDS/PAGE gel was run on venoms mixed with Laemmli sample buffer (ThermoFisher) (1 : 1) to give a final volume of 20 μL, added into individual tubes, heated at 95 °C for 5 min before running electrophoresis at 150 V for approximately 60 min.

#### Nuclear magnetic resonance (NMR) spectroscopy

NMR spectroscopy was used to observe the presence of small molecules in crude SvV and ShV. Crude venoms were pooled from multiple spines of each individual fish for analysis. In addition, seven individual spines from SvV were also analyzed to investigate any differences in venom composition between spines.

NMR data were obtained as previously reported following standard procedures [27]. Spectra were recorded at 290 and 298 K on a Bruker Avance III 600 MHz spectrometer (Bruker, Billerica, MA, USA) equipped with a cryoprobe. Crude venom samples were prepared in 90% H_2_O/10% D_2_O (*v*/*v*) (99.9%, Cambridge Isotope Labs, Tewksbury, Massachusetts, United States of America), vortexed, and centrifuged prior to transfer to an NMR tube (Wilmad, 5 mm). One‐dimensional (1D) and two‐dimensional (2D) spectra were collected using standard Bruker pulse programs, and referenced to external 4,4‐dimethyl‐4‐silapentane‐1‐sulfonic acid (DSS; Cambridge Isotope Laboratories). The 1D data included a cpmgpr1d experiment to suppress the larger peptide and protein signals. The 2D spectra included TOCSY, NOESY, COSY, HMBC, ^13^C‐HSQC, and ^15^N‐HSQC experiments with TOCSY and NOESY mixing times of 80 and 500 ms, respectively. Spectra of crude SvV were also collected in 100% MeOD (Cambridge Isotope Labs, methanol‐d_4_) to observe peaks otherwise obscured by the water suppression. Spectra were analyzed with topspin v3.6.3 (Bruker).

#### Liquid chromatography‐mass spectrometry (LC–MS)

LC–MS experiments were performed to further examine the molecular weights and venom compositions using standard procedures for LC–MS. Samples were mixed with LC–MS solvent A (H_2_O/0.1% formic acid (FA, Sigma‐Aldrich, Castle Hill, New South Wales, Australia) (1 : 2)), centrifuged, and the supernatant analyzed using a Shimadzu LCMS‐2020 mass spectrometer coupled to a Shimadzu Prominence HPLC system (Shimadzu, Kyoto, Japan). Solvent B contained 90% acetonitrile (ACN, Sigma‐Aldrich)/10% H_2_O/0.09% FA.

Crude venom samples were injected via an autosampler (Shimadzu SIL‐20ACHT) onto a RP‐HPLC column (Phenomenex Kinetex 5 μm C_8_ 100 Å 50 x 2.1 mm; Phenomenex, Torrence, CA, USA) kept at 35 °C. Solvent was delivered by Shimadzu LC‐20 AD pumps at a flow rate of 0.250 mL·min^−1^, and the absorbance monitored at 214 nm and 280 nm (Shimadzu SPD‐20A detector). Mass spectra were collected in positive and negative ion modes (scan range *m*/*z* 200–2000), with a detector voltage of 1.15 kV, and nebulizing and drying gas flows set to 1.5 and 3.0 L·min^−1^, respectively. Crude venom samples were eluted with a 1% gradient (Solvent B: 0–80% in 80 min). Data were then analyzed with the Shimadzu LabSolutions v5.96 software (Shimadzu) and graphpad prism Ver 9.

### Analytical assessment of fractions and partially purified small molecules

#### Size‐exclusion chromatography (SEC)

SEC was performed with both SvV and ShV using standard procedures with the aim of fractionating the protein components of the venom. Crude venom samples were fractionated using an Agilent 1260 Infinity HPLC (Agilent Technologies, Hanover) and a Phenomenex Yarra 3 μm SEC‐2000, 300 × 7.8 mm column (Phenomenex). Venoms were diluted (1 : 1) with DPBS and centrifuged prior to loading. An isocratic elution of 100% DPBS at a flow rate of 0.8 mL·min^−1^ for 60 min was used. Individual fractions were collected manually. SEC fractions were pooled from repeat experiments, freeze‐dried, and kept at −80 °C until they were required.

#### Solid‐phase extraction (SPE)

Solid‐phase extraction columns (Phenomenex Strata C18‐U 55 μm 70 Å) were used to partially purify the small molecules found in both SvV and ShV. The column was first equilibrated twice with 1 mL 100% methanol and washed twice with 1 mL 100% H_2_O prior to loading the venom samples. The column was then washed with 1 mL 5% methanol/H_2_O, and the partially purified small molecules were eluted with 100% methanol prior to dilution and freeze‐drying, and later rehydrated and analyzed using NMR spectroscopy.

#### NMR and LC–MS of venom fractions and partially purified small molecules

Individual SEC fractions from SvV and ShV were analyzed with NMR spectroscopy and LC–MS. NMR spectra were recorded using the methods applied to the crude venoms, and LC–MS samples were run as described above except using a reversed‐phase C_18_ column (Phenomenex Aeris, 3.6 μm PEPTIDE, XB‐C18, 150 × 2.1 mm, 100 Å) and with the inclusion of a desalting step (0% B, 10 min) followed by a 1% gradient of solvent B (0–60% B, 60 min) over a scan range of 95–2000 *m*/*z* in positive and negative modes. Data were then analyzed with TopSpin NMR software (Bruker), Shimadzu LabSolutions v5.96 software (Shimadzu) and GraphPad Prism Ver 9.

### Biological assessment of crude venoms, fractions and partially purified small molecules

#### Cytotoxicity assessment of stonefish venom in whole blood T cell lymphocytes

As fish venoms are known to possess immunomodulatory activity [28], these venoms were tested for the first time in whole blood T‐cell lymphocytes to ascertain cytotoxicity. Human blood was obtained from healthy donors and drawn by a trained phlebotomist (ethics number H6702, James Cook University Human Ethics Committee). All participants provided written informed consent. The study was carried out according to the rules of the Declaration of Helsinki of 1975. Human T‐cell lymphocytes (peripheral blood mononuclear cells [hPBMCs]) were purified from whole blood using standard procedures [29] and were either used immediately or cryopreserved. Cells that were cryopreserved were centrifuged at 400 ***g*** for 5 min to remove any medium and were resuspended in freezing buffer at the desired concentration. Cells were transferred into 1 mL cryovials (Cryo.s vials, Greiner Bio‐One) and frozen using a standard freezing device (Corning CoolCell FTS30 or LX) with a controlled freezing rate to −80 °C, and subsequently transferred to liquid nitrogen within 48 h for long‐term storage.

Cryopreserved cells were thawed and diluted in R10 medium (ThermoFisher RPMI‐1640 medium with 10% ThermoFisher FBS) in a 1 : 10 ratio, then centrifuged at 400 ***g*** for 5 min. The cell pellet was resuspended in R10 medium and treated with 20 μL of DNase‐I (Merck) at 37 °C for a minimum of 20 min to ensure DNA digestion and prevention of negative effects from dead cell debris. The cell pellet was washed twice to remove DNase at 500 *g* for 5 min and resuspended with R10 medium each time. After resuspension, an automated cell counter (OLS CASY 2.5, OMNI Life Sciences) analyzed and obtained viable cell numbers.

Lyophilised crude venom and fractions from SEC and SPE were reconstituted with DPBS at different concentrations (20–7.5 μg·mL^−1^). Briefly, hPBMCs were centrifuged and seeded at 100 000 per well in triplicate in a white flat‐bottom opaque 96‐well plate (BMG Labtech, Mornington, Victoria, Australia). Lysis buffer (positive control), DPBS (negative control), and venom treatments were then added. Fluorescence was measured at six different time points with a plate reader FLUOStar Omega (BMG Labtech) following the manufacturer's protocol (CellTox Green, Promega, Alexandria, New South Wales, Australia). Some fractions could not be tested due to insufficient sample amounts (fraction 2 from SvV and fraction 4 from ShV).

#### Electrophysiology

Due to the intense pain sting victims report and the role of sodium channels (Na_v_) such as nicotinic acetylcholine receptors (nAChRs) and the γ‐aminobutyric acid type A receptor (GABA_A_R) in pain, the influence of crude ShV and SvV on these channels/receptors was investigated. The effects of crude lyophilized ShV and SvV were tested on human neuronal (α7) and adult muscle‐type (α1β1δε) nAChRs, and α1β2γ2 GABA_A_R. For experiments on sodium channels, SEC fractions from SvV were analyzed on rNa_V_1.3, mNa_V_1.6, hNa_V_1.7, hNa_V_1.8, and hNa_V_1.9_C4. ShV was not tested due to insufficient venom amounts. Expression of sodium channels, nAChRs and GABA_A_R in *Xenopus laevis* oocytes used T7 or SP6 mMESSAGE‐mMACHINE transcription kits (Ambion, Brisbane, Queensland, Australia ). Oocytes were injected with 50 nL of cRNA (1 ng·mL^−1^) and incubated in ND96 solution with 96 mm NaCl, 1.8 mm CaCl_2_, 2 mm KCl, 1 mm MgCl_2_, and 5 mm HEPES (pH = 7.4), supplemented with 50 mg·L^−1^ gentamycin sulphate. Recordings were achieved using a Geneclamp 500 amplifier (Molecular Devices, Sunnyvale, CA, USA) and controlled by a pClamp data acquisition system (Axon Instruments, Foster City, CA, USA) with a ND96 bath solution. Voltage and current electrodes were then filled with 3 m KCl with resistance kept between 0.7 and 1.5 MΩ. Elicited currents were sampled at 20 kHz and filtered at 2 kHz with the use of a four‐pole low‐pass Bessel filter. Leak subtraction was performed using a ‐P/4 protocol. Data were obtained for three or more independent experiments.

For the α1β2γ2 GABA_A_R, cells were clamped at −70 mV. A protocol of two pulses of 10 mm γ‐aminobutyric acid (GABA, Sigma) agonist for 30 s and a 3‐min washout interval between each pulse was performed. For nAChRs, the oocytes were voltage‐clamped at a holding potential of −70 mV. Acetylcholine (ACh, Sigma) was applied via gravity‐fed tubes until peak current amplitude was obtained (1–3 s), with 1‐ to 2‐min washout periods between applications. The nAChRs were gated by a variable time‐duration pulse of ACh (10 μm for αβδε and 20 μm for α7) for the different nAChR subtypes at 2 mL·min^−1^. Data were sampled at 500 Hz and filtered at 200 Hz. Peak current amplitude was measured before and after peptide incubation. Venoms were lyophilized and reconstituted in 25 μL (ShV) and 10 μL (SvV) ND96 buffer, and 5 μL ShV and 3 μL SvV used per experiment. Samples were added directly to the bath via pipette.

For oocytes expressing Na_V_ channels, currents were evoked by a 100 ms depolarization to the voltage corresponding to the maximal activation of the channels in control conditions from a holding potential of −90 mV. Lyophilized SvV fractions were reconstituted in 500 μL ND96 buffer and 20 μL applied directly to the bath via pipette.

## Results

Venom samples from *S. verrucosa* and *S. horrida* were analyzed using SDS/PAGE, SEC, LC–MS, and NMR spectroscopy to provide insight into their molecular composition. SEC was used to fractionate the venom and the activity profile of the crude venom and fractions was assessed through cytotoxicity and electrophysiology studies.

### Molecular analysis of crude stonefish venoms

**Fig. 1:**
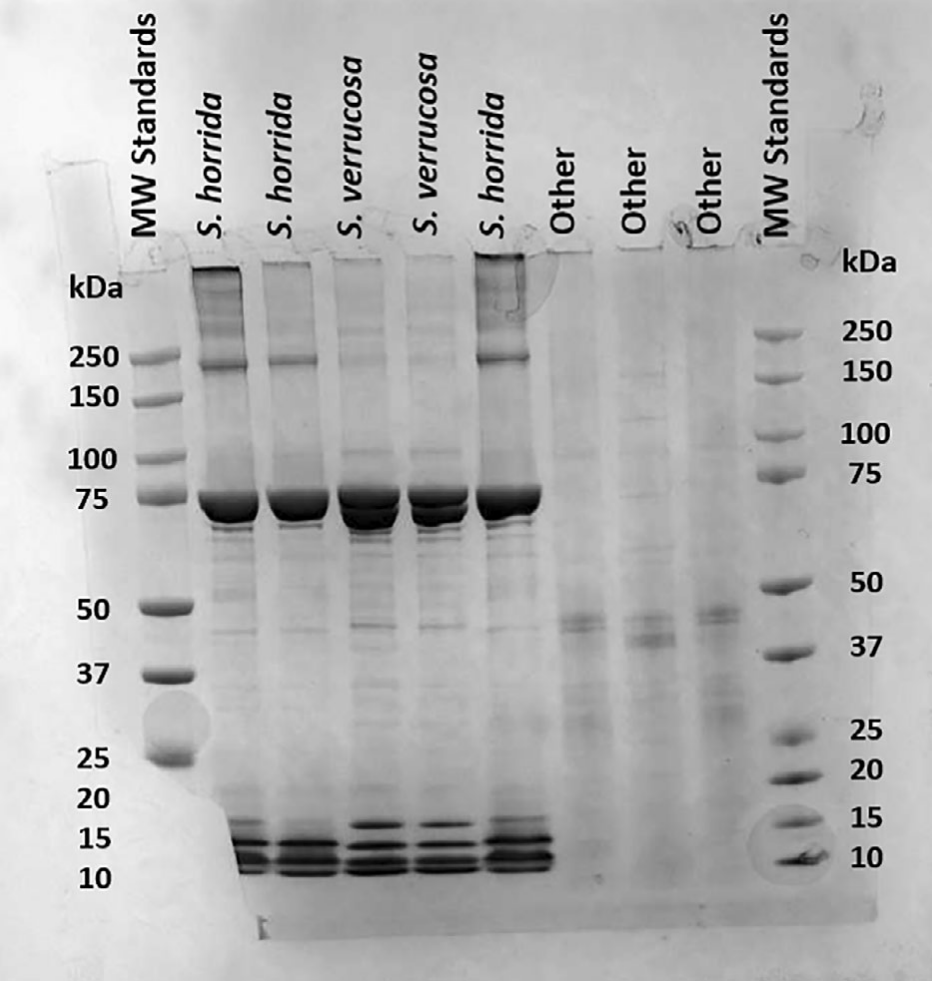
SDS‐PAGE gel of stonefish venoms. Molecular weight standards in kDa are highlighted at both ends (lanes 1 and 10). ShV in lanes 2 and 6 are technical replicates and SvV in lanes 4 and 5 are technical replicates. ShV in lane 3 is a second biological replicate. Lanes 7–9 (marked as “other”) are unrelated samples.

Analysis of the stonefish venoms using SDS/PAGE indicated that the venom is dominated by components with a molecular weight of approximately 75 kDa (Fig. 1), most likely corresponding to the monomer subunits of the verrucotoxin [14], neoverrucotoxin [30], and stonustoxin [31] proteins previously characterized. There were also several proteins with molecular weights between 10 and 15 kDa. Interspecies differences were evident, with some components at around 250 kDa present in ShV, but not in SvV, whereas SvV had darker bands around 17 and 47 kDa (Fig. 1).

**Fig. 2:**
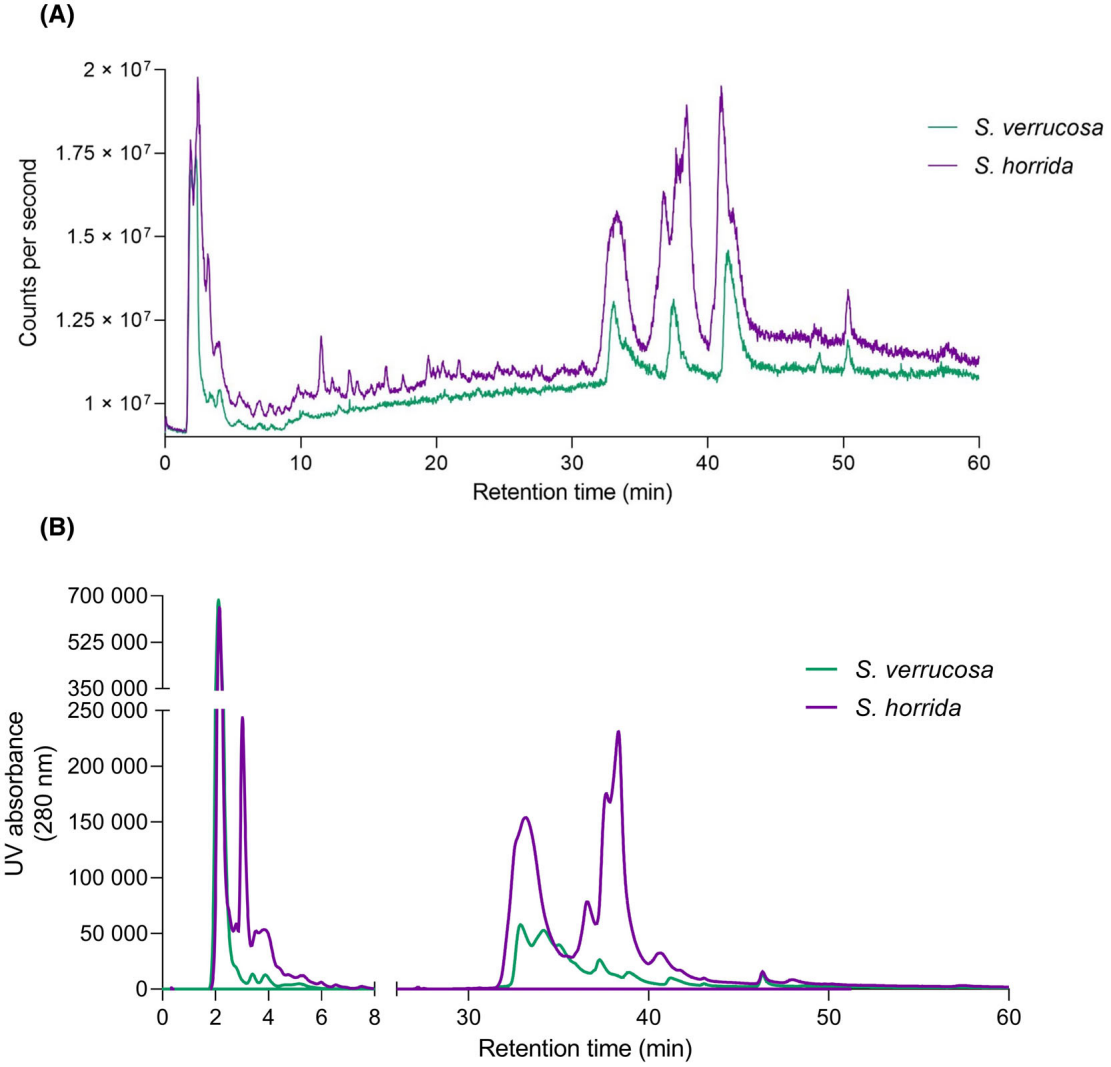
Liquid Chromatography‐Mass Spectrometry TIC and UV_280 nm_ chromatograms of *Synanceia verrucosa* and *Synanceia horrida* venoms. (A) TIC and (B) UV absorbance (280 nm). LC–MS chromatograms run on a Phenomenex Kinetex (5 μm, C8, 100 Å, 50 × 2.1 mm) column.

LC–MS analyses were also carried out with reversed‐phase C_8_ and C_18_ columns to observe separation of proteins and the relatively small‐molecular‐weight components found in the venoms. Representative LC–MS total ion current (TIC) and UV_280 nm_ chromatograms of the SvV and ShV samples are shown in Fig. 2. Crude venoms contained masses between 12 069 and 17 376 Da, consistent with the SDS/PAGE data, and also contained several small molecules ranging in molecular weight from 103 to 564 Da (Table S1). The larger proteins (> 47 kDa) were not evident in the LC–MS data.

**Fig. 3:**
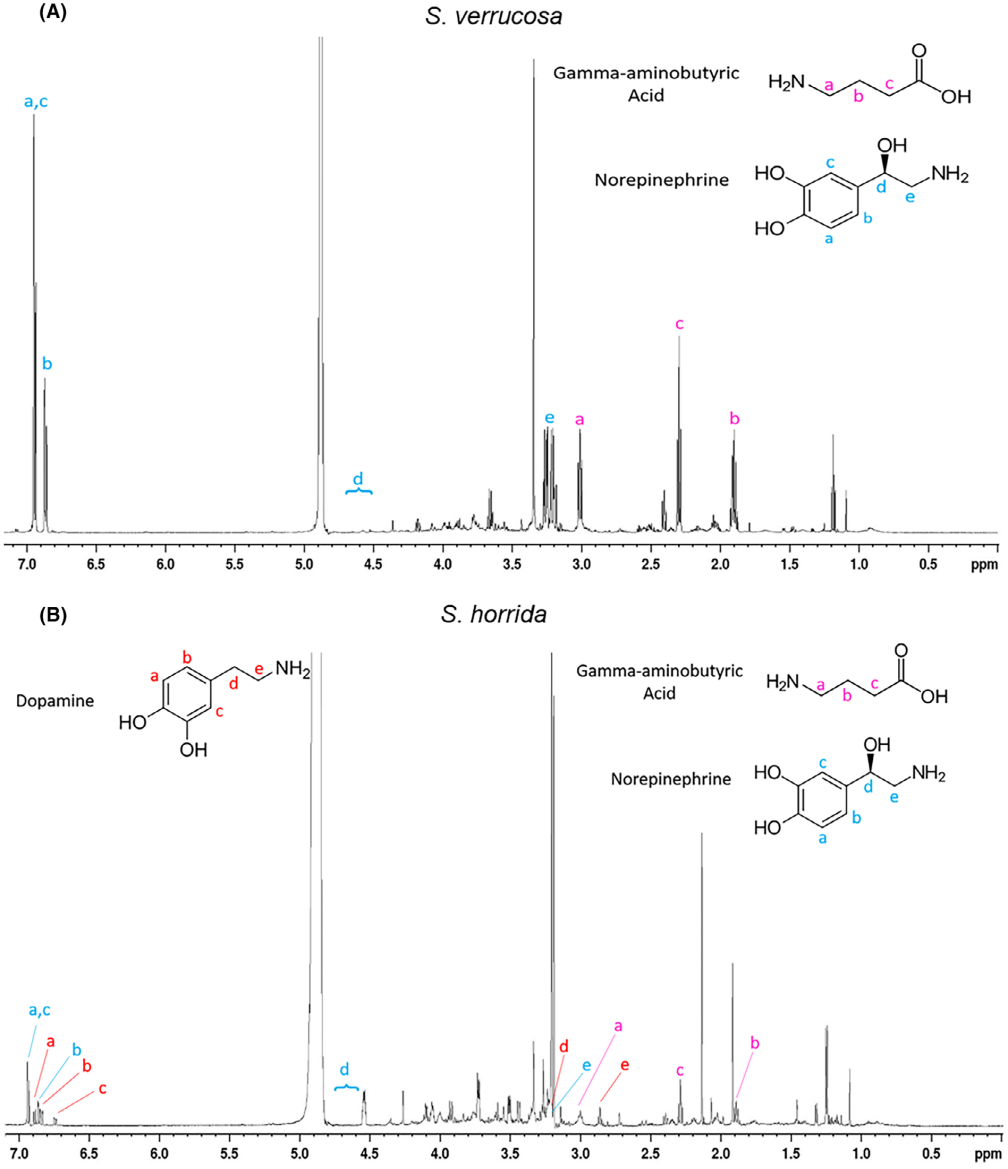
One‐dimensional NMR spectra of stonefish venoms. (A) *Synanceia verrucosa* and (B) *Synanceia horrida* frozen crude venoms showing the presence and chemical structures of norepinephrine (confirmed in both venoms), gamma‐aminobutyric acid (novel in both venoms), and dopamine (confirmed only in ShV). Peak “d” for norepinephrine at around 4.6 ppm is not evident due to water suppression, but its presence was confirmed by running the SvV sample in methanol‐d4. The assignments were derived based on ^1^H NMR spectra recorded at 600 MHz.

**Fig. 4:**
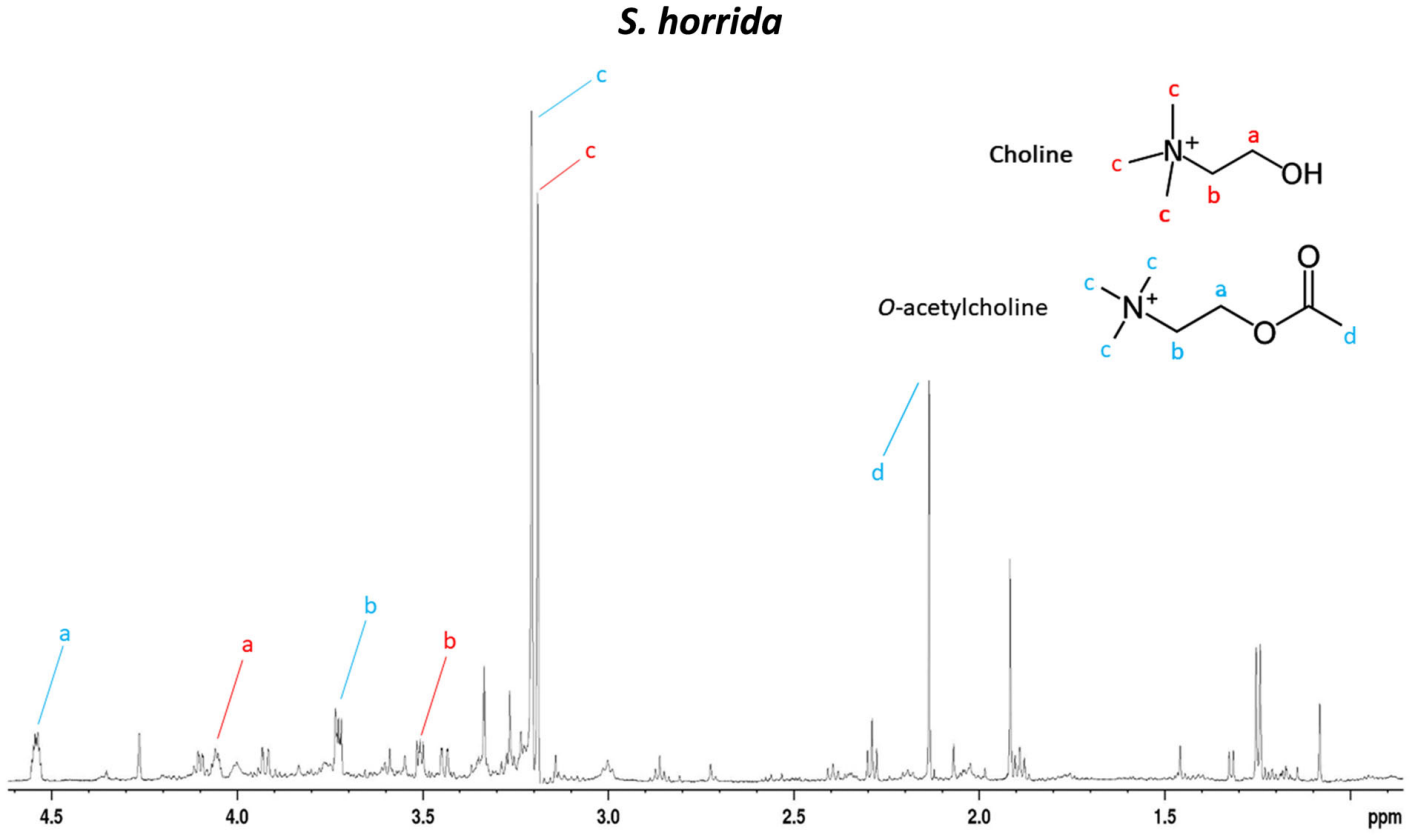
Inset of the one‐dimensional NMR spectrum from *Synanceia horrida* (Fig. 3, panel B; 0–4.6 ppm) showing the presence of choline and O‐acetylcholine. These were only observed and identified in samples from ShV. The assignments were derived based on 1H NMR spectrum recorded at 600 MHz.

NMR analysis of crude venoms highlighted the presence of several small molecules (MW 103 to 564 Da), consistent with the LC–MS data (Figs 3 and 4). Identification of the predominant small molecules within the venoms was possible based on characteristic chemical shifts and sharp peaks in the 1D spectra and interactions observed in the 2D spectra, but some less abundant components remain unidentified in both venoms.

**Fig. 5:**
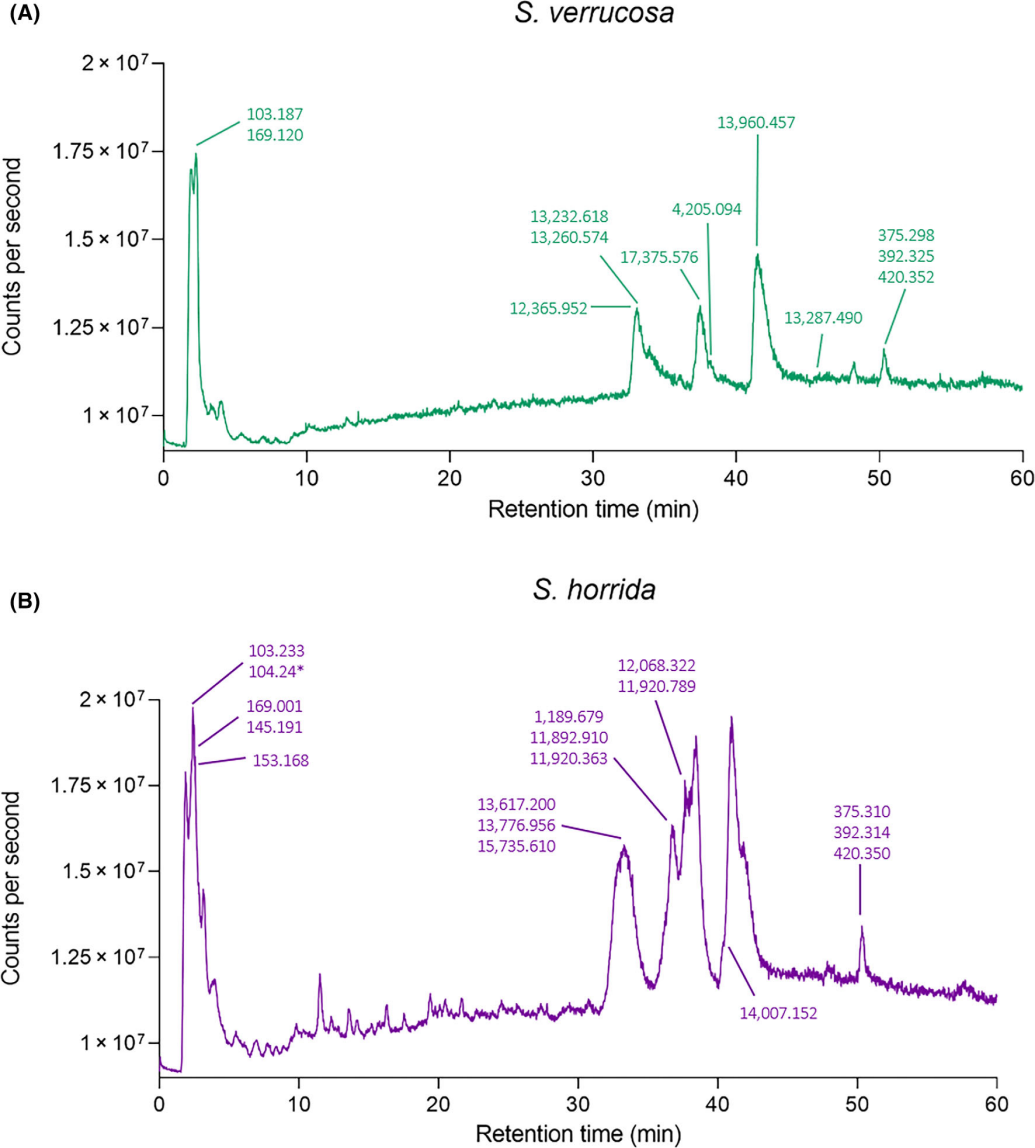
TIC Liquid Chromatography‐Mass Spectrometry chromatograms of *Synanceia verrucosa* and *Synanceia horrida* venoms in positive ion mode. (A) *Synanceia verrucosa* venom: The molecular weights of gamma‐aminobutyric acid and norepinephrine have been emphasized at 2.3 min (103.187 and 169.120 Da, respectively), and (B) *S. horrida* venom: gamma‐aminobutyric acid and choline have been emphasized at 1.7 min (103.233 Da and *m*/z* 104.24 for choline), *O*‐acetylcholine at 2.4 min (145.191 Da), dopamine at 3.2 min (153.186 Da), and norepinephrine at 2.3 min. Other molecules yet to be identified were also highlighted (Table S1). LC–MS chromatograms run on a Phenomenex Kinetex (5 μ, C8, 100 Å, 50 × 2.1 mm) column.

Crude ShV NMR analysis showed a more complex ^1^H NMR spectrum than SvV (Fig. 3). Our analysis revealed for the first time the presence of gamma‐aminobutyric acid (GABA) in both ShV and SvV (Fig. 3), and the presence of choline and *O*‐acetylcholine in ShV (Fig. 4). Norepinephrine was confirmed in both venoms, whereas dopamine could only be confirmed in ShV (not found in SvV). The presence of these molecules was confirmed by their molecular weights in the LC–MS data (Fig. 5 and Table S1). Furthermore, NMR data indicated that norepinephrine and GABA are found in approximately the same quantities (~ 1 : 1) in SvV, and norepinephrine, GABA, and dopamine are present in similar quantities (~ 1 : 1 : 1) in ShV. NMR analysis of the individual spines from SvV did not show any differences in small molecule composition between each spine.

### Molecular analysis of fractionated stonefish venom

**Fig. 6:**
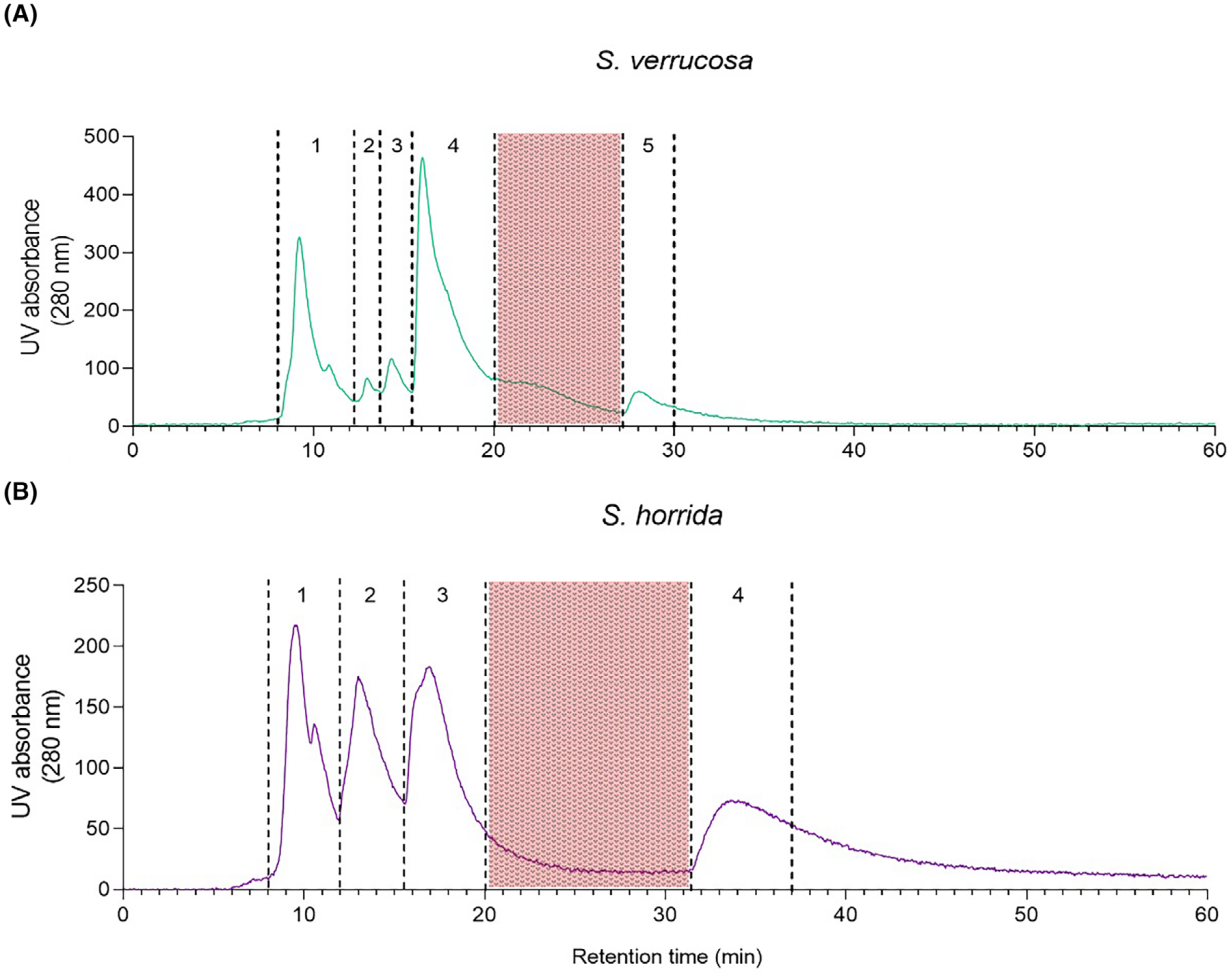
Size‐exclusion chromatography of crude stonefish venoms. (A) *Synanceia verrucosa* and (B) *Synanceia horrida* venom SEC chromatograms at 280 nm (Phenomenex Yarra 3 μm SEC‐2000 300 × 7.8 mm column). Individual fractions are numbered. Shaded areas in red were not incorporated.

Crude venoms were fractionated in DPBS using SEC. The profiles were similar for the two stonefish species, where SvV was separated into five different fractions (Fig. 6A), and ShV was separated into four fractions (Fig. 6B).

LC–MS and NMR analyses were used to investigate the composition of the individual SEC fractions of SvV and ShV. LC–MS data revealed that SEC fractions primarily contained small proteins around 11 892–17 375 Da (Table S1). NMR and LC–MS analyses of the five SEC fractions from SvV showed that fraction 1 was mainly composed of a small protein of 13 960 Da (Table S1). GABA was found in fraction 2 together with the 13 960 Da protein. Norepinephrine (NE) was found in fraction 3 along with a 17 375 Da small protein (Table S1). NE was also present in fraction 4, which also contained three other major small proteins (13 232, 13 260, and 12 365 Da). Fraction 5 was mainly composed of the 12 365 Da protein (Table S1).

Analysis of ShV SEC fractions revealed that fraction 1 was mainly composed of a 14 007 Da protein, which was also detectable in all fractions in lesser amounts (Table S1). GABA was observed in fraction 2 with two other major proteins (11 892 and 11 920 Da). These two proteins were also the main constituents of fraction 3 along with NE, dopamine, choline, and *O*‐acetylcholine. Fraction 4 contained proteins with sizes of 12 068, 13 776, and 14 007 Da. Other small molecules or proteins remain to be identified in both venoms (Table S1).

In addition, SPE was used to perform a low‐resolution separation of the crude venom to isolate the small molecules from the proteins and salts of the crude venom samples. NMR and LC–MS analyses of the SPE fractions showed a similar profile to the crude venoms, also indicating a less complex venom composition for SvV compared with ShV. Further analysis of the SPE fraction NMR spectrum indicated the presence of NE and GABA.

#### Bioactivity assessment of stonefish venoms

**Fig. 7:**
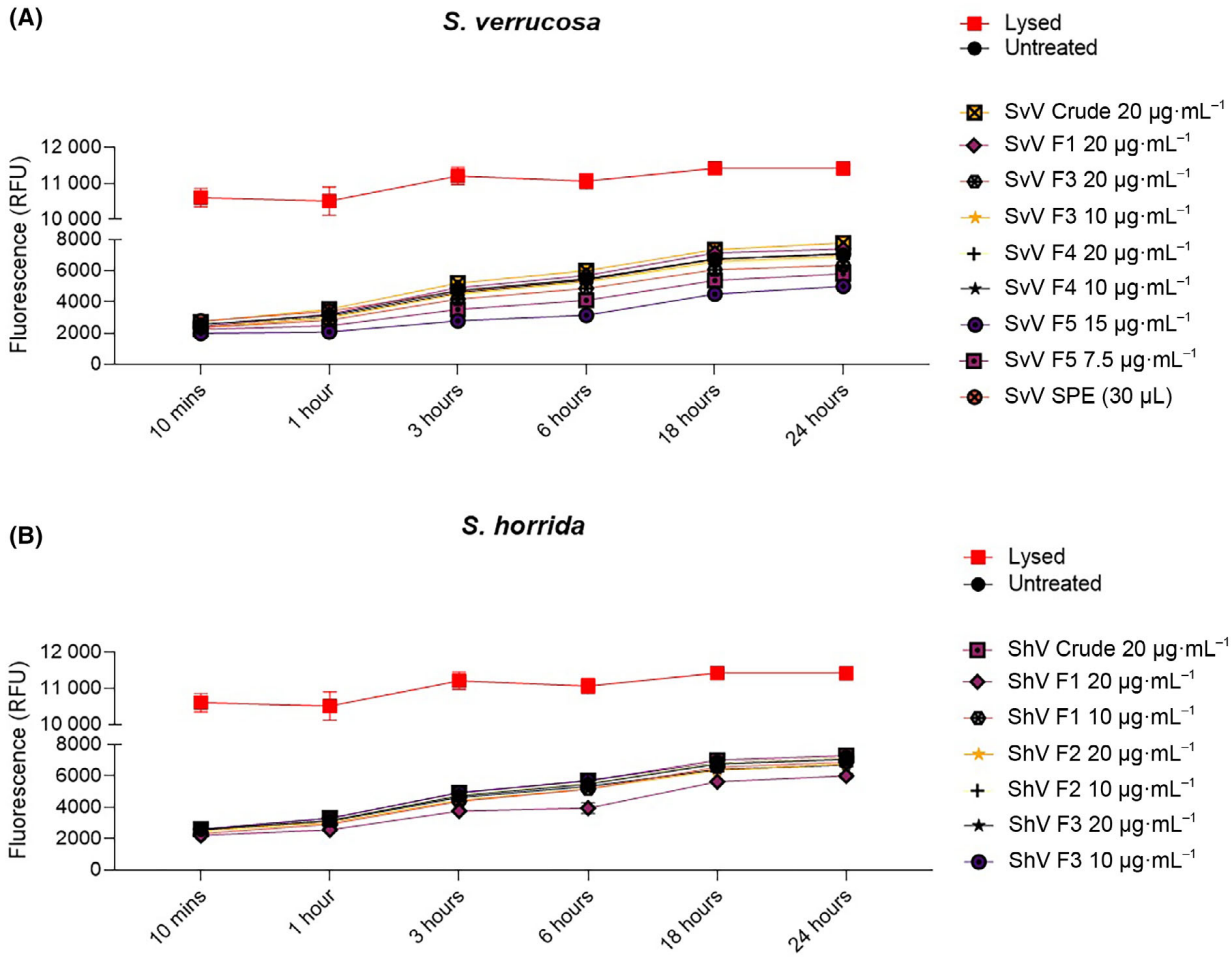
Cellular toxicity of crude stonefish venoms and respective fractions on human PBMCs. Results are the cellular response of hPBMCs to either (A) crude *Synanceia verrucosa*, SEC and SPE fractions, or (B) crude *Synanceia horrida* venom and SEC fractions. Concentrations of each fraction tested are given, but not all fractions were tested due to insufficient sample availability. Data shown as mean fluorescence ± SD of three technical replicates (*n* = 1). Lines illustrate time course of cellular toxicity measured for 24 h according to fluorescence (RFU) levels. Untreated cells are negative controls (black circle); lysed cells are positive controls (red square); venom treatments are shown in different colors.

Analysis of the cytotoxicity of the crude venom, SEC and SPE fractions was performed using a fluorescence‐based kit. Neither the crude venoms nor the fractions displayed significant cytotoxicity toward hPBMCs at the concentrations tested for either stonefish species (Fig. 7). Due to sample limitations, the cytotoxicity assay only used one biological donor and allowed for technical replicates only. Assessment of fraction 2 from SvV, or fraction 4 from ShV, was also not carried out because of limited material. Based on the cytotoxicity results from crude venom and the other isolated fractions, it is unlikely that these untested fractions would be cytotoxic toward hPBMCs.

**Fig. 8:**
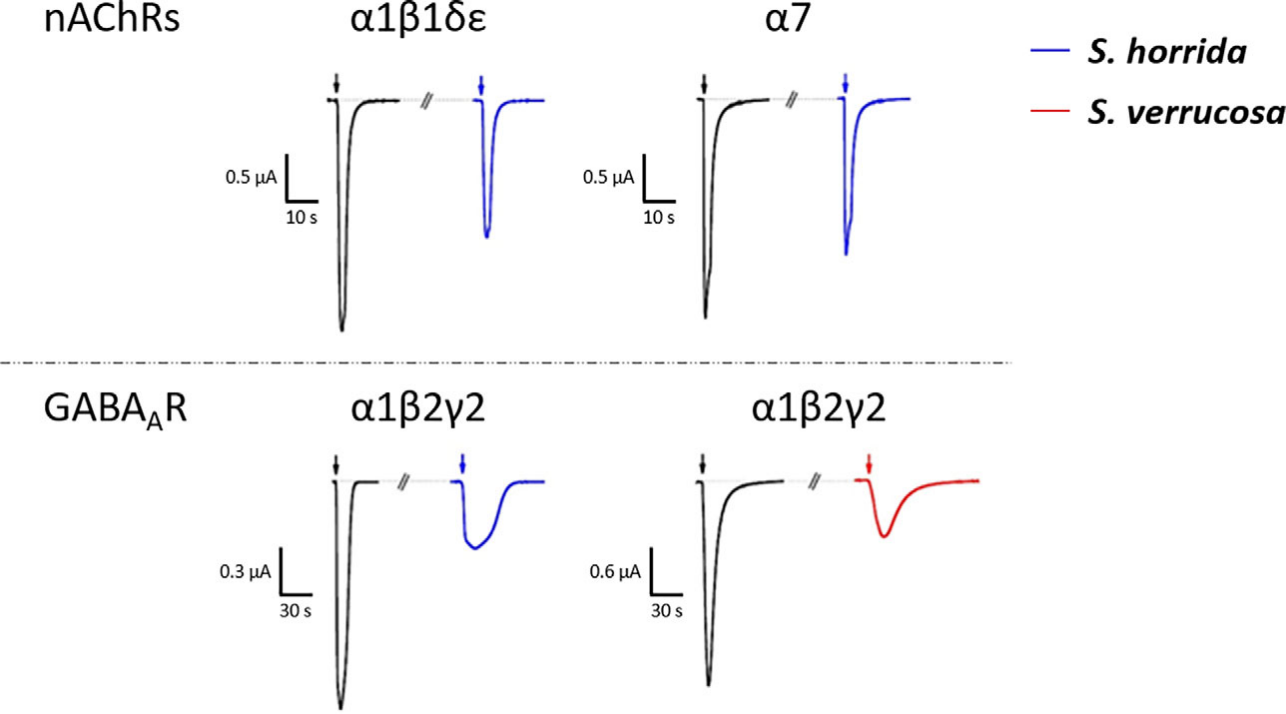
Activity of crude stonefish venoms on human nAChRs and GABA_A_Rs expressed in *Xenopus laevis* oocytes. Shown are representative traces of currents through the corresponding receptors in control (black) and after application of venom. The black arrow indicates the application of 10 μm ACh (αβδε), 20 μm ACh (α7), or 10 mm GABA (α1β2γ2). The blue arrow indicates the application of crude *Synanceia verrucosa* venom, the red arrow indicates the application of crude *Synanceia horrida* venom.

Due to the significant pain experienced in clinical symptoms of stonefish envenomation, SEC fractions from SvV were tested on voltage‐gated sodium channels associated with pain transmission [32]. Electrophysiology assays of the SvV SEC fractions on rNa_V_1.3, mNa_V_1.6, hNa_V_1.7, hNa_V_1.8, and hNa_V_1.9_C4 expressed in *Xenopus laevis* oocytes did not show any activity. Furthermore, due to the presence of ACh and GABA in the venoms, crude SvV and ShV were tested on nAChRs and the GABA_A_R. Supporting our findings, ShV activated human adult muscle‐type (α1β1δε) and neuronal (α7) nAChRs (Fig. 8). Both ShV and SvV showed activation of the human GABA_A_ receptor α1β2γ2 (Fig. 8).

## Discussion

Small molecules are often overlooked in venom analyses. This study identified some of the small molecules present in stonefish venoms. We identified choline and *O*‐acetylcholine in *S. horrida* venom for the first time, and GABA in both *S. verrucosa* and *S. horrida* venoms, which constitutes the first documentation of this neurotransmitter in fish venoms.

### Molecular analysis of stonefish venoms

Norepinephrine (NE), dopamine (DA), tryptophan (Trp), and histamine (His) have been previously reported in stonefish venoms [22, 23]. Our study confirmed NE in both venoms but did not detect Trp and His, and DA was only evident in ShV. Previously in SvV, NE was reported as the most dominant small molecule, followed by Trp and DA, respectively [23], but our results indicated that NE and GABA are present in approximately equal quantities. In ShV, DA was previously reported as the most predominant small molecule, followed by NE and finally Trp [23], but our analysis indicated that NE, GABA, and DA are present in similar quantities. This discrepancy in venom components may stem from several factors, including differences in analytical techniques and technologies used between the studies [23], and geographical or individual variations in venom composition [33]. These small molecules have also been documented in the venoms of other animals. NE and DA have been reported in a number of arthropod venoms such as bees, wasps, spiders, and scorpions [34, 35, 36, 37]. NE has also been identified in the weeverfish *Trachinus draco* [38], while *0*‐acetylcholine (ACh) has been reported in the venoms of the lionfish *Pterois volitans* [39] and the hornet *Vespa crabro* [40]. GABA, being documented here for the first time in fish venoms, has also been found in spider venoms and more recently in a wasp and a snake species [41, 42, 43, 44, 45]. Given these small molecules are present in a wide range of taxa that occupy a diverse range of habitats, we speculate they may play important roles in envenomation effects, and further investigation is warranted.

In relation to larger proteins, SDS/PAGE analysis of the stonefish venoms was consistent with previous studies, indicating that the molecular composition of the larger proteins in these two species is consistent across species, location, and time. For example, the protein bands at around 75 kDa are consistent with the size of the toxic and lethal α‐ and ß‐subunits found within both *S. verrucosa* [11, 30] and *S. horrida* [12] venoms. Furthermore, bands around 46 kDa are consistent with cardioleputin and a 45 kDa lectin found in SvV [15, 16], and bands around 9–17 kDa are also consistent with various lectin and protein sequences found in the proteome of ShV [24]. LC–MS analysis in the present study supported the results of the SDS/PAGE gel for both species, where various components ranged from 11 to 17 kDa (Table S1). The absence of the higher molecular weight proteins from the LC–MS results is likely a result of the harsh LC–MS buffer conditions (pH ~ 2 and organic solvent) and precipitation of these molecules.

The fractionation of stonefish venom using SEC and subsequent analysis by LC–MS showed unexpected elution results, with some higher molecular weight molecules eluting later than smaller molecules. Variation in hydrodynamic properties between the venom component molecules might explain some of the results observed. The presence of smaller molecules, such as GABA, in early eluting fractions with larger proteins might be the result of binding interactions between the small molecules and the proteins [46]. The SEC experiments were repeated and provided consistent and reproducible results.

### Cytotoxicity of stonefish venoms

Stonefish envenomation is well‐known for causing oedema, both locally (i.e., swelling of stung limb [47]) and/or systemically (i.e., pulmonary oedema [4]), in sting victims. For example, one case showed mild perivascular mononuclear infiltrate at the sting site that eventually led to the amputation of a toe [48]. Due to these immunological changes observed in stonefish sting victims, cytotoxicity of immunologically relevant cells was performed for the first time using stonefish venoms. The chosen cells, human peripheral blood mononuclear cells, include several classes of immune cells, such as T cells, B cells, monocytes, dendritic cells, and natural killer cells [49]. They are critical components of the adaptive and innate immune system, paramount to immunological host defense, playing roles in infection, cancer, and toxicological‐related pathologies, making them an important platform for research [49]. Since hPBMCs are paramount to the immunological response, we tested whether the venom was cytotoxic to these cells.

Stonefish venom cytotoxicity is caused by specific proteins, namely verrucotoxin and neoverrucotoxin from *S. verrucosa* venom, and stonustoxin from *S. horrida* venom, which form pores in cellular membranes [11, 26, 50]. Typically, pore‐forming toxins cause nonspecific cellular damage by binding to the plasma membrane of any cell type. The results of our cytotoxicity assay, however, suggest a cell‐specific cytotoxicity, evidenced by the fact that neither SvV nor ShV showed cytotoxicity toward hPBMCs at concentrations of up to 20 μg·mL^−1^. Although it would have been preferable to have multiple donors for this assay due to biological variation [51], our findings are consistent with previous reports showing that stonefish venom has weak toxicity toward human erythrocytes, and exhibits time‐, temperature‐, and dose‐dependent cytotoxicity effects toward HeLa cells and/or human cardiomyocytes [11, 13, 17, 52].

### Cardio physiological and neuromuscular effects of stonefish venoms

The cardiovascular and neuromuscular effects of experimental stonefish envenomation have shown highly variable results, leading to controversy. It has been suggested that cardiovascular collapse and neuromuscular blockage occur due to venom effects on adrenoceptors or muscarinic receptors, but it is unclear whether this is a direct or indirect effect [22, 53]. The presence of specific molecules such as NE, DA, GABA, ACh, and choline in stonefish venoms could potentially clarify some of the ambiguous reports found in the literature. Depending on their individual concentration and ability to penetrate surrounding tissues, these small molecules might play an important role in the cardiorespiratory and neuromuscular effects observed in both clinical and laboratory envenomation [38].

Previous studies of ShV and one of its toxic fractions (trachynilysin) tested on murine and frog neuromuscular junctions showed neuromuscular toxicity was caused by massive increase in quantal release of ACh from nerve terminals at low concentrations, and by neuromuscular damage at higher concentrations [54, 55]. Another study using ShV on rat brain synaptosomes reported venom‐driven release of ACh endogenous stores [52]. In a pharmacological assessment of ShV using longitudinal smooth muscle of guinea pigs, Hopkins *et al*. [22] concluded that venom might cause the release of ACh, substance P, or cyclooxygenase, or that venom contains cholinomimetics that act on those receptors or α_1_‐adrenoceptors. The presence of the neurotransmitter ACh and its precursor, choline, in ShV is consistent with the findings of Hopkins *et al*. and might have confounded the observed results of the previous studies due to a direct response of ShV on neuronal and muscular nACh receptors. Similarly, the venom of *P. volitans*, which contains high levels of Ach, induced muscle fibrillation and blockage of neuromuscular signal transmission through substantial release and consequent depletion of ACh from nerve terminals on frog nerve‐muscle preparations [39]. The authors concluded the presence of ACh might account for some of the cardiac physio pathologies observed in *P. volitans* experimental envenomation and that ACh might act synergistically with the venom toxins to enhance local vasodilation and/or pain [39]. This activity and synergistic action might also occur in envenomations by *S. horrida*.

Fewer studies of SvV have been undertaken and less literature is available. An assessment of fresh SvV on postsynaptic binding activity on nAChRs mimotopes (sequenced for a variety of taxa) using biolayer interferometry showed toxins in the venom might bind to nAChR directly; however, this was not confirmed [56]. Our study showed choline and ACh were not present in SvV and the venom did not stimulate neuronal or muscle‐type nAChRs, so it is currently not possible to conclude if SvV has a direct effect on nAChRs. These differences in venom composition and receptor activation between *S. horrida* and *S. verrucosa* might find future clinical use in the development of treatments specific for each species.

The catecholamines NE and DA modulate cardiorespiratory effects and their presence in stonefish venom might play a major role in stonefish envenomation. NE has important roles in the sympathetic regulation of respiratory and cardiovascular systems [57], potentially causing bradycardia [58] and producing positive chronotropic and inotropic effects [59]. In SvV experimental envenomation on frog atrial fibers and myocytes, venom caused positive and dose‐dependent chronotropic and inotropic effects [60]. DA has also been correlated with increases in cardiac output and stroke volume in healthy volunteers; however, the cardiovascular effects produced by DA were not as significant as for NE [58]. GABA is a potent inhibitory neurotransmitter and is also capable of modulating cardiovascular function [61, 62], with a range of effects including transient tachycardia, hypotension, and respiratory discomfort upon intravenous injections in mice and rats [63, 64]. In our study, both SvV and ShV activated the human α1β2γ2 GABA_A_R. The difference in the activation profiles on the α1β2γ2 GABA_A_R between the stonefish venom samples and the control GABA are likely the result of adding the small sample volume by pipette directly to the sample bath compared to the gravity‐fed addition of control GABA.

### Nociception mechanisms in stonefish envenomation

It is unclear whether the severe effects associated with stonefish envenomation are a consequence of venom‐modulated cardio physiological toxicity, venom‐driven neuromuscular collapse, or the excruciating pain that causes stress‐induced pathologies on the cardiorespiratory system [65]. The involvement of molecules such as NE could potentially play a role in venom nociception, as noradrenergic mechanisms in the peripheral tissues increase nociceptor excitability upon tissue injury, ultimately leading to pain aggravation [57, 59]. In addition, vasoconstriction due to noradrenergic effects may promote ischemic states in peripheral tissues, which may lead to ischemic pain [57]. In this study, SEC fractions from *S. verrucosa* were tested on various Na^+^ channels, some of which, such as Na_V_1.3, Na_V_1.7, Na_V_1.8 and Na_V_1.9, play a major role in nociceptive signaling [32]. No activity was observed at these channels for the *S. verrucosa* SEC fractions, which indicates that venom‐driven pain modulation is unlikely to involve these Na^+^ channels in *S. verrucosa* envenomation. An important avenue for future studies is to further investigate the molecular composition of stonefish venoms and their respective nociceptive cascade effects.

## Conclusion

This study identified the presence of novel molecules in stonefish venom and attempted to elucidate their potential implications in envenomation. In addition to GABA, ACh, and choline, many other venom constituents revealed by NMR spectrometry and LC–MS data remain unidentified. Further testing and identification of these molecules is warranted to understand the roles they might play in the venoms. Additionally, to the best of our knowledge, no study has been published on the peripheral levels of NE, DA, GABA, ACh, and choline and their mechanistic action toward cardio and neuromuscular physiology. Further study investigating the abundance of these small molecules in stonefish venoms, the implications of peripheral administration of these molecules, and the possible mechanisms of action correlating to the clinical symptoms observed is required.

The presence of GABA in earlier eluting SEC fractions together with small proteins might be the result of GABA interacting with the coeluting protein, so further investigation to characterize the protein(s), establish whether an interaction is present, and the role of the interaction is warranted. Furthermore, venoms from different stonefish species differ in composition, so a comparison and contrast of the species, with focus on the pathologies and symptoms, might show that the different species cause different pathophysiological effects. This could translate to improved and more effective treatment of sting victims, or even provide opportunities for novel drug discovery. As technologies and methodologies continue to improve, so does the ability to tease apart the intricate composition of complex venom mixtures, so it is essential to continue investigating stonefish venoms to improve treatment modalities and to explore their potential as drug leads or as physiological tools.

## Conflict of interest

The authors declare no conflict of interest.

## Author contributions

SLS wrote the paper; all authors planned and performed experiments; SLS analysed data. All authors have read, critically reviewed, and provided feedback on the manuscript. DTW and NLD: Conceptualization. DTW, NLD, SLS, SP and JT: Methodology, investigation, data curation, formal analysis. SLS: Writing—original draft. DTW and NLD: Supervision, resources. All authors have read, critically reviewed, and provided feedback on the manuscript.

## Supporting information


**Table S1.** LC‐MS analysis of stonefish crude venom components and their respective SEC fractions with the molecular weights (MW, Da) listed. Yellow highlights GABA and NE (103 and 169 Da, respectively) found in crude SvV; green highlights GABA/choline, NE, ACh and DA (104, 169, 145 and 153 Da, respectively) found in crude ShV; grey highlights the major constituents of each SEC fraction per stonefish species. RT is the retention time (min); ✓ marks the presence of the molecule; * marks *m*/*z* values (not possible to distinguish GABA from choline because they have similar *m*/*z* values despite having distinct MWs); SEC fractions are given by “F” followed by their respective numbers.

## Data Availability

The data that support the findings of this study are available from both corresponding authors (silvia.saggiomo@qimrberghofer.edu.au and david.wilson4@jcu.edu.au) upon reasonable request.
