# Supplementary material for: Interrogating stonefish venom: small molecules present in envenomation caused by Synanceia spp
Source: FEBS Open Bio. 2024 Nov 20;15(3):399–414. doi: 10.1002/2211-5463.13926 (PMC11891765; doi:10.1002/2211-5463.13926)
Supplement: Supplementary file 1 — Table S1. LC‐MS analysis of stonefish crude venom components and their respective SEC fractions with the molecular weights (MW, Da) listed. Yellow highlights GABA and NE (103 and 169 Da, respectively) found in crude SvV; green highlights GABA/choline, NE, ACh and DA (104, 169, 145 and 153 Da, respectively) found in crude ShV; grey highlights the major constituents of each SEC fraction per stonefish species. RT is the retention time (min); ✓ marks the presence of the molecule; * marks m/z values (not possible to distinguish GABA from choline because they have similar m/z values despite having distinct MWs); SEC fractions are given by “F” followed by their respective numbers. [file FEB4-15-399-s001.docx]

## Supplementary information

**Table S1: LC-MS analysis of stonefish crude venom components and their respective SEC fractions with the molecular weights (MW, Da) listed.** Yellow highlights GABA and NE (103 and 169 Da, respectively) found in crude SvV; green highlights GABA/choline, NE, ACh and DA (104, 169, 145 and 153 Da, respectively) found in crude ShV; grey highlights the major constituents of each SEC fraction per stonefish species. RT is the retention time (min); ✓ marks the presence of the molecule; * marks m/z values (not possible to distinguish GABA from choline because they have similar m/z values despite having distinct MWs); SEC fractions are given by “F” followed by their respective numbers.

|  |  |  | *S. verrucosa* - SEC | | | | | | *S. horrida* - SEC | | | | |
| --- | --- | --- | --- | --- | --- | --- | --- | --- | --- | --- | --- | --- | --- |
|  | RT (min) | MW (Da) | Crude | F1 | F2 | F3 | F4 | F5 | Crude | F1 | F2 | F3 | F4 |
| GABA/Choline | 1.7 | *104.24** |  |  |  |  |  |  | ✓ |  |  |  |  |
|  |  | 122.126 |  |  |  |  |  |  | ✓ |  |  |  |  |
|  |  | 140.097 |  |  |  |  |  |  | ✓ |  |  |  |  |
|  |  | 145.105 |  |  |  |  |  |  | ✓ |  |  |  |  |
|  |  | 163.083 |  |  |  |  |  |  | ✓ |  |  |  |  |
|  |  | 168.079 |  |  |  |  |  |  | ✓ |  |  |  |  |
|  |  | 202.247 |  |  |  |  |  |  | ✓ |  |  |  |  |
|  |  | 218.028 |  |  |  |  |  |  | ✓ |  |  |  |  |
|  |  | 236.014 |  |  |  |  |  |  | ✓ |  |  |  |  |
|  |  | 241.034 |  |  |  |  |  |  | ✓ |  |  |  |  |
|  | 1.9 | 122.116 | ✓ |  |  |  |  |  |  |  |  |  |  |
|  |  | 140.080 | ✓ |  |  |  |  |  |  |  |  |  |  |
|  |  | 145.097 | ✓ |  |  |  |  |  |  |  |  |  |  |
|  |  | 163.086 | ✓ |  |  |  |  |  |  |  |  |  |  |
|  |  | 168.077 | ✓ |  |  |  |  |  |  |  |  |  |  |
|  |  | 218.011 | ✓ |  |  |  |  |  |  |  |  |  |  |
|  |  | 236.001 | ✓ |  |  |  |  |  |  |  |  |  |  |
| GABA | 2.3 | 103.187 | ✓ |  |  |  |  |  |  |  |  |  |  |
|  |  | 151.140 | ✓ |  |  |  |  |  |  |  |  |  |  |
| NE |  | 169.120 | ✓ |  |  |  |  |  |  |  |  |  |  |
| NE |  | 169.001 |  |  |  |  |  |  | ✓ |  |  |  |  |
| ACh | 2.4 | *146.189** |  |  |  |  |  |  | ✓ |  |  |  |  |
|  | 2.6 | 154.124 | ✓ |  |  |  |  |  |  |  |  |  |  |
| DA | 3.2 | 153.186 |  |  |  |  |  |  | ✓ |  |  |  |  |
|  |  | 171.139 |  |  |  |  |  |  | ✓ |  |  |  |  |
|  | 3.3 | 307.106 | ✓ |  |  |  |  |  |  |  |  |  |  |
|  | 4.0 | 258.128 | ✓ |  |  |  |  |  |  |  |  |  |  |
|  |  | 347.088 | ✓ |  |  |  |  |  |  |  |  |  |  |
|  | 4.8 | 181.155 |  |  |  |  |  |  |  |  |  |  |  |
|  | 5.3 | 157.181 |  |  |  |  |  |  | ✓ |  |  |  |  |
|  |  | 211.137 |  |  |  |  |  |  | ✓ |  |  |  |  |
|  | 5.4 | 365.179 | ✓ |  |  |  |  |  |  |  |  |  |  |
|  |  | 389.320 | ✓ |  |  |  |  |  |  |  |  |  |  |
|  | 5.5 | 236.102 |  |  |  |  |  |  | ✓ |  |  |  |  |
|  | 6.2 | 275.108 |  |  |  |  |  |  | ✓ |  |  |  |  |
|  | 7.0 | 612.212 | ✓ |  |  |  |  |  |  |  |  |  |  |
|  | 7.9 | 208.139 | ✓ |  |  |  |  |  |  |  |  |  |  |
|  | 9.8 | 301.136 |  |  |  |  |  |  | ✓ |  |  |  |  |
|  | 10.3 | 236.105 |  |  |  |  |  |  | ✓ |  |  |  |  |
|  | 11.5 | 283.198 |  |  |  |  |  |  | ✓ |  |  |  |  |
|  | 12.3 | 287.150 |  |  |  |  |  |  | ✓ |  |  |  |  |
|  | 12.7 | 275.156 |  |  |  |  |  |  | ✓ |  |  |  |  |
|  | 12.9 | 323.089 | ✓ |  |  |  |  |  |  |  |  |  |  |
|  | 13.6 | 659.499 |  |  |  |  |  |  | ✓ |  |  |  |  |
|  | 14.1 | 312.134 |  |  |  |  |  |  | ✓ |  |  |  |  |
|  | 15.2 | 244.196 |  |  |  |  |  |  | ✓ |  |  |  |  |
|  | 15.9 | 564.373 |  |  |  |  |  |  | ✓ |  |  |  |  |
|  | 16.3 | 244.249 |  |  |  |  |  |  | ✓ |  |  |  |  |
|  |  | 285.275 |  |  |  |  |  |  | ✓ |  |  |  |  |
|  | 19.2 | 872.580 |  |  |  |  |  |  | ✓ |  |  |  |  |
|  |  | 1000.661 |  |  |  |  |  |  | ✓ |  |  |  |  |
|  | 19.8 | 315.388 |  |  |  |  |  |  | ✓ |  |  |  |  |
|  | 20.1 | 414.330 |  |  |  |  |  |  | ✓ |  |  |  |  |
|  | 20.5 | 379.232 |  |  |  |  |  |  | ✓ |  |  |  |  |
|  |  | 424.262 |  |  |  |  |  |  | ✓ |  |  |  |  |
|  | 20.9 | 317.215 |  |  |  |  |  |  | ✓ |  |  |  |  |
|  | 21.6 | 2387.436 |  |  |  |  |  |  | ✓ |  |  |  |  |
|  | 23.0 | 2810.048 | ✓ |  |  |  |  |  |  |  |  |  |  |
|  | 24.5 | 2716.891 |  |  |  |  |  |  | ✓ |  |  |  |  |
|  | 30.8 | 940.587 |  |  |  |  |  |  | ✓ |  |  |  |  |
|  | 33.2 | 12365.952 | ✓ |  |  |  | ✓ | ✓ |  |  |  |  |  |
|  | 33.6 | 13617.200 |  |  |  |  |  |  | ✓ |  |  |  |  |
|  |  | 13776.956 |  |  |  |  |  |  | ✓ |  |  |  | ✓ |
|  |  | 15735.610 |  |  |  |  |  |  | ✓ |  |  |  |  |
|  | 34.4 | 13232.618 | ✓ |  |  |  | ✓ |  |  |  |  |  |  |
|  |  | 13260.574 | ✓ |  |  |  | ✓ |  |  |  |  |  |  |
|  | 36.8 | 1189.679 |  |  |  |  |  |  | ✓ |  |  |  |  |
|  |  | 11892.910 |  |  |  |  |  |  | ✓ | ✓ | ✓ | ✓ |  |
|  |  | 11920.363 |  |  |  |  |  |  | ✓ | ✓ | ✓ | ✓ |  |
|  | 37.5 | 17375.576 | ✓ |  | ✓ | ✓ | ✓ |  |  |  |  |  |  |
|  | 37.7 | 12068.322 |  |  |  |  |  |  | ✓ |  |  |  | ✓ |
|  |  | 11920.789 |  |  |  |  |  |  | ✓ | ✓ |  |  |  |
|  | 38.2 | 4205.094 | ✓ |  |  |  |  |  |  |  |  |  |  |
|  | 41.1 | 14007.152 |  |  |  |  |  |  | ✓ | ✓ | ✓ | ✓ | ✓ |
|  | 41.8 | 13960.457 | ✓ | ✓ | ✓ | ✓ | ✓ |  |  |  |  |  |  |
|  | 45.7 | 13287.490 | ✓ |  |  |  | ✓ |  |  |  |  |  |  |
|  | 50.2 | 375.310 |  |  |  |  |  |  | ✓ |  |  |  |  |
|  |  | 392.314 |  |  |  |  |  |  | ✓ |  |  |  |  |
|  |  | 420.350 |  |  |  |  |  |  | ✓ |  |  |  |  |
|  | 50.4 | 375.298 | ✓ |  |  |  |  |  |  |  |  |  |  |
|  |  | 392.325 | ✓ |  |  |  |  |  |  |  |  |  |  |
|  |  | 420.352 | ✓ |  |  |  |  |  |  |  |  |  |  |
